# Lactoferrin: Cytokine Modulation and Application in Clinical Practice

**DOI:** 10.3390/jcm10235482

**Published:** 2021-11-23

**Authors:** Santiago Presti, Sara Manti, Giuseppe Fabio Parisi, Maria Papale, Ignazio Alberto Barbagallo, Giovanni Li Volti, Salvatore Leonardi

**Affiliations:** 1 Pediatric Pulmonology Unit, Department of Clinical and Experimental Medicine, University of Catania, 95121 Catania, Italy; santiagopresti@gmail.com (S.P.); saramanti@hotmail.it (S.M.); gf.parisi@policlinico.unict.it (G.F.P.); mariellapap@yahoo.it (M.P.); leonardi@unict.it (S.L.); 2 Department of Drug Sciences, University of Catania, 95121 Catania, Italy; ignazio.barbagallo@unict.it; 3 Department of Biomedical and Biotechnological Sciences, University of Catania, 95121 Catania, Italy

**Keywords:** lactoferrin, cytokine, immune system, iron, anti-inflammatory, immunomodulation

## Abstract

Multiple properties of lactoferrin have been reported in the literature so far. Decades of in vitro and in vivo studies have demonstrated the important antimicrobial, anti-inflammatory, anti-oxidant, and immunomodulating properties. It suggests the use of lactoferrin as an effective and safe option for the treatment of several common disorders. Herein, we show the applications of lactoferrin in clinical practice, highlighting its evidence-based capacities for the treatment of heterogeneous disorders, such as allergic, gastrointestinal, and respiratory diseases, and hematologic, oncologic, gynecologic, dermatologic, and dental disorders. Moreover, the widespread use of lactoferrin in neonatology is summarized here. As a result of its antiviral properties, lactoferrin has also been proposed as a valid option for the treatment for COVID-19 patients. Here, the uses of lactoferrin in clinical practice as a new, safe, and evidence-based treatment for many types of disorders are summarized.

## 1. Introduction

**Figure 1:**
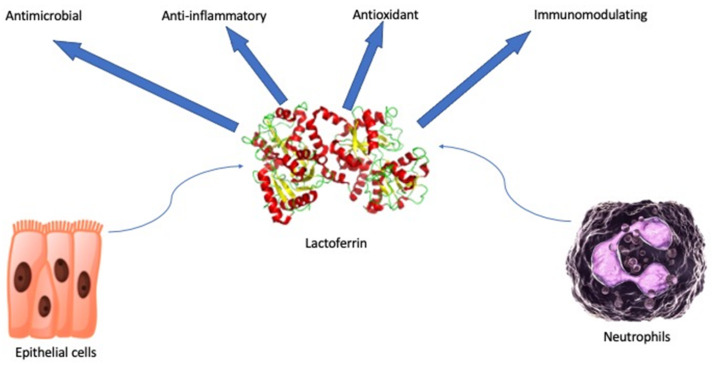
Schematic representation of pleiotropic and beneficial effects of secreted lactoferrin.

Lactoferrin (lf) is a glycoprotein of about 690 amino acid residues. It belongs to the transferrin family and it is capable of reversibly chelating two Fe(III) per molecule with high affinity (Kd ~ 10–20 M), holding ferric iron to pH values under 3.0. Differently, transferrin retains iron to pH values of about 5.5 [1]. The glycoprotein is folded into homologous N- and C-terminal lobes (Figure 1). Each lobe includes two domains, which contain a conserved iron-binding site. Interactions between the lobes influence the iron binding and release [2]. Glandular epithelial cells and neutrophils following induction express and secrete human lactoferrin (hlf). The highest levels can be found in human colostrum (~7 g/L) [3]. Hlf and bovine lactoferrin (blf) share high sequence homology and multiple functions: antimicrobial (antibacterial, antifungal, antiviral, and antiparasitic properties), anti-inflammatory, antioxidant, and immunomodulating [4] (Figure 1). Antimicrobial effects are several: bacteriostatic properties are performed by the lf iron sequestration with a reduction of microbial and biofilm growth and bactericidal capabilities are carried out by the lf interaction with the Gram-negative lipopolysaccharide (LPS) and the lipothecoic acid of the Gram-positive bacteria. Serine protein protease activity of lf and direct inhibitory effects on viruses have also been demonstrated [5].

Several other immunological and systemic properties are described in the literature, endowing lf as a new safe and effective natural player in clinical practice of several common diseases. Herein, as shown in Table 1, we report the most updated clinical applications of lf.

### Cytokine Modulation and Immunological Effects

Since the early 2000s, several studies have highlighted the capacity of lf in modulating the immune response. Artym et al. demonstrated that bovine lf improved the immune response in immunocompromised mice treated with cyclophosphamide in 2003 and with methotrexate in 2004 [6,7]. In 2005, the same authors found that mice that had received lf after a treatment with busulfan and cyclophosphamide had less inhibited cellular immune responses. Thus, in bone marrow transplant, enhanced lympho-, erythro-, and myelopoiesis has been demonstrated [8]. Lf possesses many properties in modulating the innate and acquired immune system [9,10]. It interacts with the immune system in several ways. Specifically, an increase of the percentage of natural killer cells and modulation of T and B cells and antibody formation has been demonstrated [11]. Studies highlight that cytokine activity is also influenced by lf, either upregulation of anti-inflammatory cytokines, such as IL-4 and IL-10, or modulation of proinflammatory ones, such as TNF-a, IL-1, IL-6, and granulocyte-macrophage colony-stimulating factor [12]. An increase of the levels of IL-6 with a subsequent enhancement of bacteria clearance was demonstrated by Artym and colleagues [13]. Lf also influences the release of chemokines, such as IL-8. It affects the growth, differentiation, activation, and functions of immune cells [14]. Moreover, studies demonstrated CD4+ T cell differentiation with an increase of the Th1/Th2 cytokine ratio with a burst of the Th1-type immune response and higher expression of Th1-type cytokines, such as IFN-γ and IL-12 [15,16]. As a natural consequence, allergic reactions, normally modulated by a Th2 response, can be downregulated [17]. The ability of lf to inhibit eosinophil migration takes part in the downregulation of allergic inflammation [18]. Lf binds bacterial components, such as LPS, flagellin, and nucleic acids, inhibiting the inflammatory response. Thus, immunological effects are modulated by the lf interaction with receptors (Toll-like receptors [TLRs], CD 14 receptor) on gut-related epithelia with lower proinflammatory signal. Lf activates dendritic cells (DCs), promoting the release of IL-12 by DCs and downgrading the IL-4 secretion by Th2 cells. Controversially, apoptotic death of Th1 cells is counteracted by lf. As a result, the response against microbic attacks is enhanced [19]. Lf stimulates the proliferation of human peripheral blood mononuclear cells and signal pathway modulations are described, including induction of the CD4 antigen and lymphocyte adhesion molecule-1. An increased expression of the zeta chain in the CD3 complex, leading to the transmission of the activation signal in T cells, has been reported [20]. Proinflammatory cytokine responses to pathogens are also reported by the effects of human Lfcin (hLf1-11) peptide. It leads to the activation of TLR-4, -5, and -7 signal pathways with the upregulation of intracellular NF-κB, leading to a proinflammatory cytokine response to pathogens [21]. In murine models, porcine lf decreased TNF-α serum levels and the mRNA expression of innate markers of inflammation in the intestine. In fact, a reduction of monocyte chemotactic protein-1 (MCP-1), macrophage inflammatory protein (MIP)-1α, IL-6, TNF-α, and IFN-γ has been demonstrated. Moreover, an increase of the anti-inflammatory levels of IL-10 is important to highlight [22]. Activation and phagocytosis of polymorphonuclear leukocytes (PMNs) and monocytes/macrophages are enhanced by lf. In fact, studies have revealed that lf is a promoter of superoxide production, motility, and release of proinflammatory molecules, such as NO, TNF-α, and IL-8 [13]. Due to the aforementioned heterogenous properties, lf has multiple clinical applications as we show below.

## 2. Materials and Methods

We focused on the evidence-based findings that highlight the efficacy of lf for the treatment of several common disorders. PubMed was searched using the search term “lactoferrin”. Only English meta-analyses, randomized controlled trials, and clinical trials were filtered.

Intervention studies involving animals or humans, and other studies that required ethical approval, must have listed the authority that provided approval and the corresponding ethical approval code.

## 3. Results

The following results highlight the effects and outcomes related to the therapy with lf.

### 3.1. Lactoferrin in Neonatology

The majority of studies about the use of lf in newborns are focused on preventing neonatal sepsis and necrotizing enterocolitis (NEC). In 2009, Manzoni et al. were the first who demonstrated a reduction of the incidence of late-onset sepsis (LOS) in preterms with birth weight under 1500 g, using lf alone and/or in combination with Lactobacillus rhamnosus GG (LGG) [23]. Again, in 2014, Manzoni and his co-workers evidenced that the use of blf supplementation alone or in combination with LGG reduced the incidence of ≥stage 2 NEC and of death and/or ≥stage 2 NEC in preterms under 1500 g [24]. After the aforementioned studies, a lot of other RCTs demonstrated the efficacy of lf in neonatal intensive care units. The most updated evidence is shown in a Cochrane meta-analysis that involved more than 5200 preterm neonates [25]. The study highlighted the efficacy of lf for the prevention of late-onset sepsis (both bacterial and fungal sepsis) with or without probiotics. According to this study, lf also prevents urinary tract infections and decreases the length of hospital stay. In this work, the prevention of NEC stage II or III has been demonstrated using the association of lf with probiotics and not by the use of lf alone. A recent meta-analysis by Yi He and colleagues suggests that the use of lf in preterms slightly shorts the time required to achieve full enteral feeding (FEF), reduces the incidence of hospital-acquired infections, and reduces the infection-related mortality [26]. Thus, the administration of lf in very low birth weight neonates counteracts the higher risk of sepsis induced by the use of gastric acid inhibitors [27]. As suggested by Pammi and Gautham, it is correct to start prophylaxis as soon as possible with a dosage of 150–300 mg/kg/day (based on birth weight) [25]. It is important to highlight that no adverse effects have been reported so far and it has been demonstrated that the use of lf is safe and well-tolerated [28,29]. It is important to mention the protective mechanism of lf in preterm developmental brain hypoxic damage. According to Ochoa and Sizonenko, it derives from various factors, such as downregulation of proinflammatory cytokines, secretion by activated microglia, iron toxicity reduction, oxidative stress reduction, neuronal differentiation factor expression, support of brain development, and promotion of neurotrophic factors’ expression [30]. Neuroprotective effects have been demonstrated in mice, which showed less lipopolysaccharide-mediated brain injury [31] and better cerebral gray and white matter recovery after cerebral hypoxia-ischemia [32]. All the aforementioned features suggest the use of lf for the prevention of encephalopathy in preterms.

### 3.2. Lactoferrin and Respiratory Viruses

Several RCTs report the efficacy of lf in respiratory illness. Infants aged 4–6 months fed with lf-fortified formula milk (38 mg/100g milk) showed a reduction of morbidity of respiratory infections. Specifically, this group of patients had lower episodes of rhinorrhea, cough, and wheezing [33]. Respiratory benefit was also reported by King et al., in particular, a group of healthy formula fed infants >34 weeks’ gestation and >4weeks of age received formula cow milk supplemented with lf (850 mg/L) [34]. At one year follow-up, compared to a placebo control, the lf group showed fewer incidences of lower respiratory tract illness with significantly lower episodes of wheezing. The decrease of the incidence of colds and its related symptoms has been demonstrated by the use of a combination of lf/whey protein Ig-rich fraction (lf/IgF) 600 mg/daily in a cohort of adult patients, suggesting this association as a preventive strategy [35]. In 2011, Lang et al. demonstrated that lf inhibits the entry of Severe Acute Respiratory Syndrome Coronavirus (SARS-CoV) into HEK293E/ACE2-Myc cells and blocks the binding of spike protein [36]. Consequently, several studies suggested the use of lf for the treatment of SARS-CoV 2 [37]. In particular, in vitro studies proved the antiviral activity of lf against COVID-19 due to the direct attachment to both SARS-CoV-2 and the cell surface, suggesting the potential role in the management of COVID-19 [38]. Campione et el. proposed a trial using lf either oral or solubilized intranasal spry administration against the pandemic virus [39]. They suggested that this treatment option might be considered in asymptomatic or paucisymptomatic patients to avoid the worsening of SARS-CoV2. The few studies that enrolled patients showed a shortening of the conversion of the rRT-PCR SARS-CoV2 RNA and lower IL-6 and D-Dimer levels, and a decrease of the length of symptoms has been highlighted [40]. It is important to mention that neither RCTs nor meta-analysis are currently available and further research is needed in order to better define the role of lf in the management of the current pandemic.

### 3.3. Lactoferrin and Gastrointestinal Disease

Evidence seems to demonstrate that lf can ameliorate the symptoms and severity of gastroenteritis in children. The prophylactic use of 1000 mg of lf per day in patients between 12 and 18 months old seems to shorten the duration of diarrhea and to reduce episodes with moderate or severe dehydration [41,42]. Rotavirus gastroenteritis seems to be ameliorated by the use of a prophylactic therapy with 100 mg/daily of lf in children under 5 years old, showing a reduction of the frequency and duration of vomiting and diarrhea [43]. Recombinant hlf (1 g/L) has been administrated in association with lysozyme (ly) (0.2 g/L) in a rice-based oral rehydration solution in children with acute diarrhea and dehydration. Patients showed a significant decrease in the duration of symptoms, faster reaching of solid stool, less volume of diarrhea, and less children had new episodes of liquid stools after resolution [44]. Antiparasite activities of lf have been highlighted by Ochoa et al. when studying the prevalence and incidence of diarrhea in a group of children aged 12–36 months using blf supplementation [45]. At 9 months follow-up, they found no significant differences in the incidence and prevalence of diarrhea compared with the placebo group. Nonetheless, the lactoferrin group had fewer prevalence of Giardia species colonization and better growth, highlighting the antimicrobial properties of this glycoprotein against these kinds of parasites. Favorable effects on gut health have been demonstrated by Cheng et al. using lf 1.5 g/day and lysozyme 0.2 g/day supplementation [46]. After 16 weeks, in a population of 12–23-month-old children, compared to the placebo group, there were lower amounts of hospitalization and the development of acute malnutrition. In adults, lf seems to prevent acute gastrointestinal symptoms as shown by Mizuki et al. Three groups of adults received lf 600 mg/day, 200 mg/day, or nothing for 12 weeks. The groups who received lf showed lower prevalence of acute gastroenteritis, lower duration of diarrhea, as well as cumulative prevalence days of abdominal pain, nausea, diarrhea, and fever [47]. Lf could also be effective in the treatment of post-antibiotic diarrhea [48].

### 3.4. Lactoferrin and Onco-Hematologic Disorders

Lf’s safety and capacity to interact with iron has meant that it has been long used in patients with anemia, as demonstrated in a group of at-term Kenyan infants, aged 6–9 months with anemic status [49]. The group received for 3 months an intervention of a fortification of lf (76 mg/100 g) to the iron-fortified formula. After 3 months, this group, compared to the placebo one, showed significantly higher levels of hemoglobin (Hb). As shown in this study, it is important to mention that after 1 month, there was no difference in the Hb concentration between the blf and placebo group, suggesting that the efficacy of blf depends on the intervention time and it should be more than 1 month [50]. In 2016, Rezk et al. showed that in pregnant women (second trimester) affected by iron deficiency anemia, the use of lf was more effective than ferrous sulfate [51]. Specifically, Hb after 2 months of treatment was 2.26 ± 0.51 g/dL higher, compared to ferrous sulfate (1.11 ± 0.22 g/dL). The study highlighted that gastrointestinal adverse events occurred more frequently in patients treated with ferrous sulfate. As a consequence, more women treated with ferrous sulfate requested to change the drug with the other one. Paesano and co-workers demonstrated in a group of pregnant and non-pregnant women that the administration of 100 mg twice/day of blf improved the hematological parameters more than the use of ferrous sulphate 520 mg/day. Specifically, in pregnant women, blf significantly decreased serum IL-6 and increased prohepcidin. Instead, in non-pregnant women, prohepcidin was increased. This suggests that blf is a safe treatment and it is more efficient than ferrous sulphate at treating iron deficiency and iron deficiency anemia in pregnant and non-pregnant women [52]. Cancer patients may have complications affecting various organs [53]. In cancer patients receiving chemotherapy, the supplementation of lf showed an improvement in taste and smell abnormalities: patients who experienced the aforementioned symptoms showed higher levels of salivary Fe with a reduction of salivary immune proteins [54]. Specifically, 1-month supplementation of 750 mg of lf significantly decreased the concentration of salivary Fe, and increased the salivary α-amylase and the immune proteins. After the treatment, the taste and smell abnormalities score was significantly reduced [54]. Other studies demonstrated a correlation between higher immune activity and suppression of colorectal polyps. In patents aged 40 to 75 years with polyps ≤ 5 mm, the use of 3 g daily of blf for 12 months significantly retarded adenomatous polyp growth. It suggests that lf use could be a valid therapy in addition to polyp extraction [55]. It can be explained by the role of lf in the modulation of the immune system. In fact, as already mentioned, the increased activity of NK cell, serum hlf levels (indicating increased neutrophil activity), and numbers of CD4+ cells in polyps have been highlighted [56].

### 3.5. Lactoferrin and Allergic Disorders

As already mentioned, lf downregulates allergic inflammation with a lower Th2 response and eosinophil migration, with a consequential decrease of allergic reactions, as demonstrated in allergic airway inflammation in asthma [57,58]. Lf, together with Ly, is the most important antimicrobial and anti-inflammatory protein of the upper respiratory tract. As demonstrated, patients affected by chronic rhinosinusitis with nasal polyposis show lower lf levels [59]. Wang et al. investigated the effect of intranasal administration of hlf in mice with allergic rhinitis (AR), showing a reduction of Th2 inflammation and symptoms, suggesting that the use of lf might be effective for the prevention and treatment AR [60]. A decrease of allergic airway inflammation has also been demonstrated by Kruzel et al. in cultured bronchial epithelial cells in a mouse model [61]. Specifically, a lower accumulation of eosinophils in airways and lower cellular oxidative stress levels has been demonstrated, suggesting the use of lf in allergic inflammatory disorders. Studies highlight that topical lf inhibits the allergen-induced mobilization and migration of epidermal Langerhans cells in humans, normally activated after skin sensitization [62]. Similarly, in a double-blind phase 2 study, Tong et al. found that the use of a combination of lf and bovine whey-derived Ig-rich fraction led to beneficial effects in patients with atopic dermatitis (AD), demonstrated by an improvement of the SCORAD and DLQI scores [63]. Taken together, the aforementioned findings suggest that lf might play a safe and effective role in the treatment of allergic disorders.

### 3.6. Others

An oral combination of Lactobacillus acidophilus GLA-14, Lactobacillus rhamnosus HN001, and blf has been used in women with vulvovaginal candidiasis (VVC), demonstrating an improvement of itching and discharge at 3 and 6 months compared with the placebo group [64]. Moreover, the use of the same combination in women with bacterial vaginosis has been associated with a decrease of symptoms, such as vaginal discharge and itching [65]. Additionally, an improvement of dermatological lesions in patients with acne vulgaris has been evidenced by Kim and co-workers using 200 mg of lf daily for 12 weeks. In particular, a reduction of inflammatory lesion count by 38.6%, total lesion count by 23.1%, and acne grade by 20.3% were highlighted. Thus, a reduction of 31.1% of the sebum content was found [66]. Similar results were shown by Chan et al. lf is also an important host defense factor of saliva [67]. Studies have demonstrated that the intake of lf (60 mg/d) and lactoperoxidase (another host defense found in saliva) (7.8 mg/d) improved gingival inflammation and oral health-related quality of life after a treatment of 12 weeks [68].

## 4. Conclusions

Lactoferrin is a defense glycoprotein that fulfils a crucial task in the natural host defense showing antimicrobial, anti-inflammatory, antioxidant, and immunomodulating properties. It is found in the secretions of exocrine glands and is one of the elements secreted by neutrophils. In this work, as summarized in Table 1, we showed the wide use of lf in clinical practice and its evidence-based benefits. We examined the role, efficacy, and safety of this glycoprotein in neonatology, pulmonology, gastroenterology, allergic disorders, onco-hematology, dermatology, gynecology, and dentistry. Taken together, the aforementioned studies strengthen the choice of lf as a new, safe, and powerful treatment against a widespread range of diseases.

## Figures and Tables

**Table 1:** Applications of lactoferrin in clinical practice.

Area	Results
Neonatology	Prevention of LOS, urinary tract infections, decreased length of hospital stay. Prevention of NEC stage II or III if associated with probiotics. Less time to achieve FEF. Fewer incidence of hospital-acquired infections. Reduction of infection-related mortality
Pulmunology	In infants: fewer incidence of lower respiratory tract illness. Prevention of rhinorrhea, cough, and wheezing. In adults: lower incidence of cold-related symptoms
Gastroenterology	In children: amelioration of symptoms and severity of gastroenteritis, less days of vomiting and diarrhea. Lower prevalence of Giardia species colonization. In adults: prevention of acute gastrointestinal symptoms. Efficacy in post-antibiotic diarrhea.
Allergic disorders	Reduction of Th2 inflammation, lower accumulation of eosinophils, and decreased cellular oxidative stress levels in mice with allergic airway inflammation. In humans, topical inhibits allergen-induced mobilization and migration of epidermal Langerhans cells. Improvement of the SCORAD and DLQI scores in patients with atopic dermatitis
Onco-Hematology	Improvement of Hb in anemic children and pregnant women. Improvement of taste and smell abnormalities in patients receiving chemotherapy. Retardation of adenomatous polyp growth
Dermatology	Improvement of acne vulgaris lesions
Gynecology	Improvement of symptoms in women with VVC and bacterial vaginosis
Dentistry	Improvement of gingival inflammation
